# Let's put a smile on that face—A positive facial expression improves aesthetics of portrait photographs

**DOI:** 10.1098/rsos.230413

**Published:** 2023-10-25

**Authors:** Christian Valuch, Matthew Pelowski, Veli-Tapani Peltoketo, Jussi Hakala, Helmut Leder

**Affiliations:** ^1^ Faculty of Psychology, University of Vienna, Vienna, Austria; ^2^ Vienna Cognitive Science Hub, University of Vienna, Vienna, Austria; ^3^ Huawei Technologies Oy (Finland) Co. Ltd, Tampere, Finland

**Keywords:** image beauty, facial attractiveness, individual differences, cultural context, artificial intelligence

## Abstract

In today's age of social media and smartphones, portraits—such as selfies or pictures of friends and family—are very frequently produced, shared and viewed images. Despite their prevalence, the psychological factors that characterize a ‘good’ photo—one that people will generally like, keep, and think is especially aesthetically pleasing—are not well understood. Here, we studied how a subtle change in facial expression (smiling) in portraits determines their aesthetic image value (beyond a more positive appearance of the depicted person). We used AI-based image processing tools in a broad set of portrait photographs and generated neutral and slightly smiling versions of the same pictures. Consistent across two experiments, portraits with a subtle smile increased both spontaneous aesthetic preferences in a swiping task as well as improving more explicit aesthetic ratings after prolonged viewing. Participants distinguished between aspects associated with image beauty and the depicted person's attractiveness, resulting in specific interactions between variables related to participant traits, image content, and task. Our study confirms that a subtle—and in this case fully artificial—smile reliably increases the aesthetic quality of portraits, illustrating how current image processing methods can target psychologically important variables and thereby increase the aesthetic value of photographs.

## 1. Introduction

Never before have so many people taken so many photos—of themselves, of friends, loved ones, of trips and experiences they wanted to remember and share with others [1]. Current mobile phones make it easy for everyone to capture photos with high image quality and shape how we approach and use photography [2]. As a result, portraits are one of the most common categories of photos we encounter today [3,4], and it can be assumed that we perceive many more digital images of people, than we encounter real people. One of the criteria to evaluate images of people is beauty—the aesthetic appeal of the image, as well as of the depicted person. However, despite this increasing pervasiveness of portrait photography, surprisingly little research has looked into contextual and psychological factors that make up an aesthetically appealing photo.

A better understanding of the critical variables leading to more positive evaluations of portrait photography would allow us to explain why certain pictures are considered particularly beautiful, and to predict which pictures will have the strongest impact on their viewers. This would also have significant secondary implications and allow us to exploit the positive aesthetic effects of portrait photos in the best possible way in effective communication via social media, in public relations, or in job application processes [3,5].

Based on previous work in the fields of empirical aesthetics and art perception [6,7], we recently proposed a taxonomy of factors that determine the aesthetics of mobile phone photography [1]. Our model predicts that the perceived beauty of an image results from an interplay of image and content features that differ for different image genres and tasks, but also depends on variables of individual raters, such as their gender, expertise, familiarity with mobile phone cameras, and even their personality. Here, for the first time, we apply this framework to the genre of portrait images and investigate how the raters' gender, their familiarity with this type of image, and their expertise with photography interact with psychologically important image content variables such as the emotional expression, ethnicity and gender of the person depicted in the portrait. We study the interplay of these variables in different task contexts, which themselves might map to common ways how we encounter portrait photos—immediate, spontaneous assessments within fractions of a second, and more considered evaluations requiring longer periods of viewing—in our everyday life.

### 1.1. How do we perceive pictures of faces?

Although little empirical research has directly investigated image aesthetics in portrait photography, there is a large body of research on facial attractiveness, which usually presents participants with face portraits and asks them to rate the attractiveness of the depicted person (e.g. [8–10]). Human faces are among the most ecologically relevant stimuli for social behaviour and the perceived beauty of faces is of particular relevance to social interaction [11]. For example, looking at attractive faces is rewarding and elicits positive emotions [12–14]; beautiful faces not only attract but also bind attention [15–17]. Accordingly, our brains contain specialized neural structures and networks for processing subtle but relevant visual cues in faces with high precision [18,19]. Faces have a special status in visual perception and are processed preferentially in many ways. For example, faces that people assess as more beautiful attract and bind the viewers’ attention, even involuntarily [15,17], which could in turn influence how an overall image containing a face is evaluated.

To a certain extent, the factors associated with facial attractiveness might also predict whether a portrait image is perceived as beautiful (cf. [20]). However, the degree to which viewers are able to separate the perceived attractiveness of the person from an aesthetic assessment of the image as more or less beautiful is not yet clear (but see [21]). Therefore, it is of theoretical interest whether people can uncouple their aesthetic evaluation of an image from the evaluation of the depicted faces or persons—depending on what the current task context requires. Hence, a major goal of our current study was to investigate the extent to which judgements of image beauty and the depicted person's attractiveness can be disentangled. Accordingly, we examined if factors known to influence facial attractiveness judgements have similar or dissociable effects on explicit, aesthetic image evaluations. To that end, we produced a well-controlled stimulus set in which we manipulated psychologically relevant variables that have been previously studied in the context of facial attractiveness.

From a psychological perspective, the emotional expression of faces is highly relevant for social coexistence and mutual understanding [22–24]. Research also suggests a strong link between emotional expression and facial attractiveness: faces appear more attractive when they display a positive expression [25–28]. This might also be among the reasons why it is, nowadays, so common that people ‘smile for the camera’ or mimic a smile by ‘saying cheese’ when their picture is taken (cf. [29]). While in the early days of photography, the portrayed persons mostly showed a neutral facial expression (as it was common in paintings of that time), this practice changed with the advent of affordable and user-friendly cameras such as the Kodak camera and the consequent rise in popularity of amateur photography over the first half of the twentieth century [29]. A systematic analysis of high-school yearbook photographs showed that a smiling facial expression has become increasingly common since the early twentieth century, with an even more pronounced change in female portraits than in male ones, possibly also reflecting how cultural norms change over time [30].

However, it is currently unknown whether a positive facial expression only increases the perceived attractiveness of the depicted person or whether it also increases the aesthetic appreciation of the picture itself, which would mean that a psychologically relevant variable determines the perceived aesthetic quality of a photograph. Previous studies on the relationship of emotional expression and attractiveness often used rather strong differences between emotion conditions, for example, comparing widely smiling faces (with an open mouth) against relatively unexpressive neutral faces. This is interesting because the literature distinguishes between posed and real (or ‘Duchenne’) smiles [31], which differ with respect to which muscles and face regions are involved, and it has been suggested that posed smiles (involving only muscles around the mouth region) do not have the same evaluative benefits as real smiles [32]. For images taken with mobile cameras, there is an increasing number of filters, applied while or after the image is taken [33], that change specific features with the goal to increase beauty of the image. These beautifications comprise changes of lighting and contrast, but specifically for faces, also modifications in skin tone and texture (e.g. [34,35]). In the context of portrait photography, it would thus be particularly interesting if a subtle manipulation of emotional expression that is limited to the mouth region only, could increase the perceived beauty of the picture. In the present study, we tested if post-processing the facial expression depicted in a portrait could also increase its aesthetic value—namely through the effect of subtle, positively evaluated smiles.

The aesthetic appreciation of images could be based on shared, largely universal beauty preferences that were often linked to rather bottom-up or basic image properties and tap into basic perceptual processing mechanisms (e.g. [36–38]), which are fairly common to all humans [39]. However, cultural differences exist even for rather basic visual properties such as colour preferences (e.g. [40–42]), and the cultural context can interact with differences such as gender identity to shape which aspects contribute to an individual's preferences when evaluating faces (e.g. [43]).

For example, faces are categorized rapidly with respect to their apparent gender which modulates subsequent evaluations of attractiveness [44]. Pictures of faces also elicit differential neural processing and evaluative judgements depending on the participants' preferred gender (e.g. [45,46]). Studies of gaze behaviour (e.g. [16,17,47,48]) found that participants look longer at faces that they consider attractive, especially if they belong to the preferred gender. Face gender might also moderate the effects of emotional expression on attractiveness judgements. Tracy *et al*. [49] suggested that friendly facial expressions increased attractiveness in female faces but could have opposite effects in male faces.

The visual features that are particularly important for beauty evaluations can also differ with cultural backgrounds (e.g. [50,51]). For example, participants from the United Kingdom and China appeared to have different colour preferences when judging the attractiveness of faces ([40]; see also [52,53], for similar results). In cross-cultural studies, the agreement between different raters was higher for faces of one's own ethnicity compared with faces of other ethnicities [54]. Hence, familiarity with the facial features (cf. [55] that are prevalent in one's own cultural environment [51] could also influence how faces are evaluated [10,56,57]. Therefore, preferences can differ between individuals, as the aesthetic sense is shaped through familiarity and expertise [15,58].

### 1.2. Aims of the present study

In the present study, we go beyond isolated variables and take a comprehensive perspective on how image-, person-, and task-related variables interact to jointly determine aesthetic evaluations of portraits. Instead of assuming that picture quality largely hinges on having an up-to-date camera that produces photos with a high resolution, we assume that the psychological variables in the present paper are critical for image beauty. Specifically, we investigate how facial expression, face category (here, gender and ethnicity), and individual differences between viewers affect aesthetic evaluations of portrait images under two task contexts.

According to our model [1], viewers mostly make two types of aesthetic assessments when looking at photographs. First, viewers swipe through images on their phones or mobile devices and make spontaneous aesthetic decisions about them. Often, after just a few hundred milliseconds, they have already processed key visual features of the image that allow them to immediately and intuitively select or reject images (cf. [59–61]).

Second, individuals might stop at certain images and examine them in more detail, which can result in more deliberate thoughts and evaluations (cf. [62,63]), and involve top–down processes such as memories and associations with the image or a matching of the image content against specific task goals. People might evaluate images quite differently, depending on whether they are looking at pictures of a cocktail party they attended with their friends or looking through profile pictures of strangers in a dating app. In addition, individual differences between viewers, their gender, or personality aspects, such as their aesthetic sensitivity, could influence how they evaluate images.

First, we assessed intuitive aesthetic preferences in a swiping task, where portraits were presented only briefly, and participants had to quickly assess which images they would ‘keep’ in their picture collection. Second, in an explicit image beauty rating task, participants considered each image for six seconds before evaluating its beauty explicitly on a 7-point rating scale. In these two tasks, we instructed participants to evaluate the beauty of the image independent of the attractiveness of the person. Third, we also asked participants to explicitly rate the attractiveness of the depicted person on a different 7-point rating scale. These different tasks aimed to test if participants evaluate portraits differently, depending on the viewing duration and task goal. Based on our model [1], we assumed that these different tasks differ in their sensitivity to different factors relevant for the aesthetic experience. Specifically, we assume that the swiping task is more sensitive to fast and intuitive preferences that come within the first second after image onset, whereas explicit beauty ratings, after the image has been viewed for several seconds, are more influenced by later cognitive processes, memories and perhaps comparisons with own personal experiences.

If the aesthetic sense is a shared, largely universal mode of evaluation, we would expect different participants to prefer the same images and image features, with perhaps similar results in different evaluation tasks. Alternatively, if aesthetic judgements reflect individual and idiosyncratically evolved preferences (cf. [64]), participants should differentiate more strongly according to their own aesthetic preferences and beauty standards, which could vary between task contexts and participants.

If domain-specific factors shape aesthetic judgements, participants could flexibly take different aspects of the same images into account depending on the goals of their evaluations. For example, we would expect them to consider the image as a whole (including background, colours or framing) when evaluating its overall beauty but focus more strongly on the features of the depicted person when evaluating attractiveness. Accordingly, we hypothesized that aspects related to the depicted person, such as their gender or ethnicity, should play a larger role in attractiveness evaluations than in judgements of image beauty. In this context, the general familiarity with the face categories might be important, as our study was conducted in Europe, where most participants should be most familiar with European faces, which could also increase preferences. If, on the other hand, participants do not differentiate between faces of different genders or ethnicities, we could assume that this aspect is relatively unimportant in a specific situation, and participants might focus more on other features of the image.

To better understand the participants' individual aesthetic experiences, we asked them to describe in their own words which aspects of the images they considered important in the different evaluation tasks. We also asked them about their familiarity and experience with portrait photography, and assessed their aesthetic sensitivity using a standardized test [65] to investigate if individual differences in these variables would modulate aesthetic preferences for portraits. Our study, for the first time, goes beyond purely technical aspects or isolated variables and provides a systematic investigation into the factors that determine the perceived aesthetic quality of portraits by taking a broad set of the psychologically relevant image-, viewer- and context-related variables into account [1].

## 2. Methods

### 2.1. Participants

#### 2.1.1. Sample size

In total, 124 participants, recruited through a database at the University of Vienna, took part in the experiments. Twelve participants with a mean age of 23.8 years (*s.d.* = 3.21) completed a pilot experiment to validate the stimulus set, whereas the remaining participants were equally distributed to two main experiments (both *N* = 56; mean age of 23.5 [*s.d.* = 3.99] years in Experiment 1 and 24.2 [*SD* = 5.19] years in Experiment 2). In all experiments, half of the participants identified as male and the other half as female. All participants were naive with regard to the stimuli and hypotheses. Participants received a monetary compensation (€10 for the pilot experiment and €8 for Experiments 1 or 2).

#### 2.1.2. Statistical power

We based our sample size planning on the theoretically relevant effects of facial expression and the interactions of participant gender, face gender and facial expression. We assumed medium effect sizes (*f* = 0.25) and correlations around *r* = 0.5 between repeated measures, and used G*Power [66] to calculate the minimum sample size for different levels of statistical power. We found that, under these assumptions, a total sample size of 34 could achieve 80% power to detect main effects of the stimuli, and two-way interactions between the experimental factors, and 56 participants could achieve 95% power to detect such effects. In addition, we ensured the effectiveness of our experimental manipulations (i.e. reliable discriminability between the face categories and facial expressions) in a pre-study with independent raters (cf. §2.3.3). Before starting Experiment 2, we also verified that 56 participants should achieve 95% power to replicate the observed effects of Experiments 1. To further increase statistical power and minimize the chances of missing any potentially relevant but smaller effects, we also ran a control analysis on the combined datasets from both experiments (see electronic supplementary material, S3). Based on these considerations, we would argue that the statistical power should be adequate to answer our main research questions.

#### 2.1.3. Difference between Experiments 1 and 2

Experiment 1 and Experiment 2 were highly comparable, both utilizing the same stimuli and image assessments, with a minor methodological distinction. In Experiment 1, participants concurrently rated the perceived beauty of the image and the attractiveness of each person depicted (within a single task block). Conversely, in Experiment 2, the ratings of image beauty and person attractiveness were obtained through two distinct task blocks. Experiment 2 was primarily implemented to replicate the outcomes of Experiment 1 in a new participant sample and under a slightly altered task context.

### 2.2. Apparatus

All experiments were programmed and conducted using the LabVanced framework for online experiments [67]. Participants completed the online experiment at home using their own laptops or desktop computers. They were instructed to ensure a quiet working environment, to sit comfortably at a table and maintain the same working position and screen viewing distance over the duration of the experiment.

### 2.3. Stimuli

#### 2.3.1. Source images

The source images were colour portraits of female- and male-looking persons that appeared to be of European or Asian ethnicity with a resolution of 1024 × 1024 pixels. The images were created by a generative adversarial network (StyleGAN2, [68]) that had been trained on a dataset containing 70 000 high-quality face pictures of various age, ethnicity, and image backgrounds (https://github.com/NVlabs/ffhq-dataset). As none of the generated faces depicted an actually existing person, participants were expected to be generally unfamiliar with the generated face identities. We picked 160 different source face images that were free of any unnatural or salient visual artefacts and relatively clear examples of female-, male-, European- or Asian-looking persons. The source images featured a relatively neutral expression.

#### 2.3.2. Manipulation of facial expression

**Figure 1.**
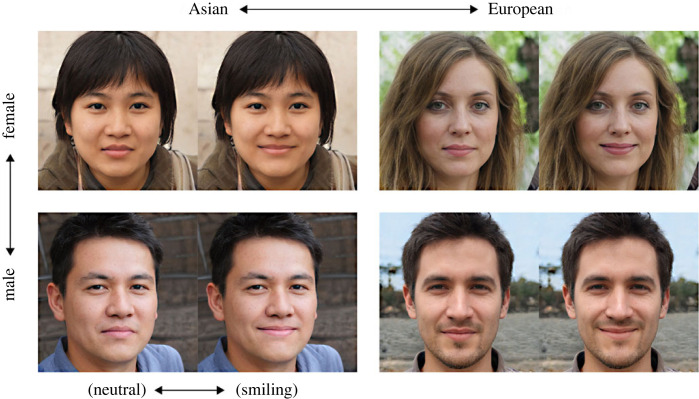
Examples of the stimuli and experimental conditions in the current study. Source images were created with a generative adversarial network trained on 70 000 portraits of different genders, ethnicities, ages, looks and image backgrounds. Each source image was processed using the Neural Filters Workspace in Adobe Photoshop to create two versions from each image, one with a neutral facial expression and one with a slightly smiling facial expression. The figure shows examples of the four categories of portraits used in the current study, in both the neutral and smiling versions.

From each source image, we created two different versions—one neutral and one smiling version—using the Neural Filters workspace in Adobe Photoshop. This software tool allows the manipulation of the emotional expression (and other features) of a face in a photorealistic fashion, based on a cloud-based image processing model that had been trained on many different face exemplars. We aimed for a subtle manipulation to avoid overly large differences in visual saliency between the two image versions and therefore kept the lips closed in all versions of the images. We selectively applied the manipulation to the mouth region, and avoided changes to other facial features in order to keep both versions as similar as possible (see example images in figure 1). The manipulated images were downscaled to a resolution of 600 × 600 pixels for use in the online experiments.

#### 2.3.3. Final stimulus selection/pilot experiment

A pilot experiment was conducted (*N* = 12 participants, see above for demographics) to validate the effectiveness of the facial expression manipulation (e.g. did participants perceive the individual to be smiling) and to verify that the individual face portraits were reliably recognized as female/male, and European/East Asian. All pictures were rated in random order. A portrait was presented centrally on the screen along with a 7-point facial expression rating scale ranging from ‘neutral’ to ‘positive’ below each picture. Afterwards, participants were presented with the smiling and neutral versions of the same faces next to each other and asked to select the image with the ‘more positive facial expression’ using the left/right arrow keys. Finally, all pictures were evaluated for gender and ethnicity on 7-point rating scales rating from ‘male’ to ‘female’, and from ‘Asian’ to ‘European’, respectively.

Most pictures were judged by participants to be relatively clear exemplars of the intended categories of male/female and Asian/European persons. A small number (*n* = 10 or 6.25%) of the initial source pictures appeared to be less typical (z-scores between 0 and ±0.5 on one of the scales, after standardization), and were excluded. From the remaining exemplars, we chose 20 from each of the four categories (smiling/neutral × Asian/European, figure 1) which showed the most substantial rating differences between the smiling and neutral versions of the same portraits and for which participants were highly accurate when choosing the more positive expression when presented side-by-side. Hence, for Experiments 1 and 2 we used 80 different identities in two emotional expression versions.

### 2.4. Experiments 1 and 2

#### 2.4.1. Design and procedure

The picture evaluation tasks in Experiments 1 and 2 were conducted in a mixed design with the between-participant factor participant gender (female, male) and with the within-participant factors of facial expression (neutral, smiling), gender of the face (female, male) and ethnicity of the face (Asian, European). To make the facial expression manipulation less obvious, participants saw each portrait only in one of the two versions—either in the smiling or in the neutral version. However, an equal number of portraits from each experimental condition was judged by every participant. The assignment of portrait versions was counterbalanced across participants so that every version received an equal number of evaluations. The main dependent variables were the proportion of ‘keep’ decisions in the swiping task, the ratings of image beauty and the ratings of person attractiveness for each evaluated portrait.

Both experiments followed the same format. After general instructions on the procedure of the study, participants entered their demographic data. Before each task, participants received written instructions (provided in electronic supplementary material, S1).

**Figure 2.**
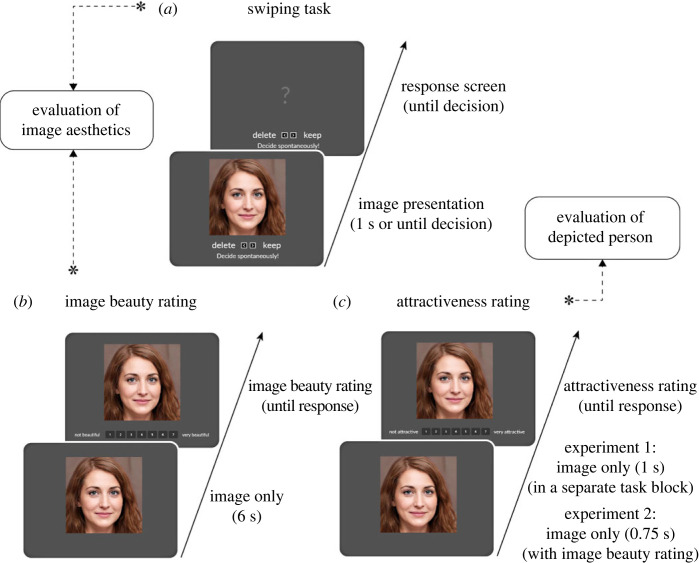
Overview of the three different evaluation tasks in the current study. (*a*) Swiping task to measure spontaneous aesthetic preferences. Each image was presented for a maximum of 1s and participants spontaneously decided whether they would delete or keep the image by quickly pressing one of two keys. (*b*) Image beauty rating to measure explicit aesthetic judgements after prolonged viewing. Each image was first presented for six seconds and participants were instructed to let the images sink in during this time. Then, a 7-point rating scale from 1 = not beautiful to 7 = very beautiful appeared below the image, and participants entered their assessment of image beauty by mouse click. (*c*) Attractiveness rating of the depicted person. To investigate if and to what extent participants distinguished between the aesthetics of the image and the attractiveness of the person depicted, participants were asked to make a separate attractiveness judgement for each image on a scale from 1 = not attractive to 7 = very attractive. In Experiment 1, attractiveness was queried in a separate task block after all pictures had already been rated once for image beauty. In Experiment 2, image beauty and attractiveness were rated in the same task block.

First, participants completed the swiping and image beauty rating tasks. In the rating tasks, participants were shown every portrait image and rated the beauty of the overall image and the facial attractiveness of the person, both on 7-point rating scales. In the swiping task, participants were shown every image for a maximum of 1 second and were asked to decide quickly and intuitively whether they would keep or delete this image. The swiping task aimed at assessing fast, spontaneous aesthetic preferences. The order of the swiping and rating tasks was counterbalanced across participants. Next, participants completed a questionnaire containing items on the aspects they found important when evaluating the images as well as items about their familiarity and expertise with photography. Participants were also asked to which degree they could separate between the dimensions image beauty and facial attractiveness. In addition, they provided information on their socio-sexual orientation on a voluntary basis. At the end, participants completed a short version of the visual aesthetics sensitivity test (VAST; [65,69]) which presented them with two similar monochromatic pictures and asked them to decide which one looked better from an aesthetic perspective. Participants typically needed between 30 to 40 min to complete the online session (figure 2).

#### 2.4.2. Tasks and questionnaires

##### 2.4.2.1. Swiping task

Before starting, participants were presented with brief instructions (see electronic supplementary material, S1) for the task together with an overview of all images in reduced size on one page to give them an idea about the variability in the stimulus set. They were asked to imagine that they had to decide quickly whether to keep in their picture collection, or delete it. The task thus required intuitive decisions similar to the ones that are made when swiping through a stack of images on a mobile phone [1]. We intentionally kept the instructions to this task very brief since we wanted to measure genuinely spontaneous decisions. We did not present participants with a more elaborate cover story or fictional situation to avoid any unintentional priming of participants on particular aspects of the material which could steer them away from their personal intuitive preferences.

Half of the participants received the instruction to press the left arrow key to ‘keep’ the image and the right arrow key to ‘delete’ the image. For the other half of the participants, the key mapping was reversed. Importantly, they were instructed to base their decision on how beautiful the image was rather than on the attractiveness of the depicted person. All pictures were presented on a black screen background. Each trial started with a central, white fixation cross (edge length equivalent to 1.5% of the screen width) for 1s. Next, a portrait image appeared for a maximum duration of 1s. The dimensions of the squared portrait image were equivalent to 50% of the screen width. If participants pressed a response button while the image was on screen, the trial ended. Otherwise, the image disappeared after 1s and was replaced by a central, gray question mark, indicating to the participants that they needed to respond. After the participants responded, the next trial started after a blank inter-trial-interval of 0.5s. An illustration of the key mapping was presented as a memory aid at the bottom of the screen, together with the hint to decide spontaneously (German: ‘Entscheiden Sie spontan!’) throughout the task block. Each participant judged 80 different portrait images in random order. The mouse cursor was hidden during the swiping task to avoid potential distractions.

##### 2.4.2.2. Image beauty rating

The explicit image beauty rating task aimed at assessing evaluative judgements after prolonged viewing. Participants first saw an overview of all the images in reduced size on one page to give them an idea about the variability in the stimulus set. They were informed that each image would be presented for six seconds, after which they should rate the beauty of the image. They were also instructed that they should base their judgements on the beauty of the image and not so much on the facial attractiveness of the depicted person. Each trial started with the central fixation cross for 1s (dimensions and colours were equal to the swiping task). Afterwards, a single portrait image was shown at the central position screen for 6 s. After 6 s, the image remained on screen, and a rating scale appeared below it with seven buttons labelled 1 through 7 (with the anchors ‘not beautiful’ and ‘very beautiful’ next to buttons 1 and 7, respectively). Simultaneously with the rating scale, the mouse cursor became visible (it was hidden during the initial viewing period to avoid potential distractions). Participants rated the perceived image beauty of the 80 portrait images (in random order) by clicking with the left mouse button on the respective button.

##### 2.4.2.3. Attractiveness judgements

For the assessment of the attractiveness of the depicted persons, participants rated each picture on a scale from 1 = not attractive to 7 = very attractive. To test whether the mode in which attractiveness was assessed influenced the dependence or independence of judgements about the beauty of the picture and the attractiveness of the person, we varied the method slightly between the two experiments. In Experiment 1, attractiveness was assessed in a separate task block after all images had already been assessed for image beauty. Here, each image was first shown for 1 s, after which the rating scale appeared, and participants made their judgements. In Experiment 2, attractiveness was always rated immediately following the image beauty rating within the same task block: after entering the rating of image beauty, the first scale disappeared, and the image remained alone on the screen for 0.75 s before the attractiveness rating scale appeared, and participants entered their attractiveness judgement for the same image.

##### 2.4.2.4. Post-evaluation questionnaires

*Familiarity.* To assess the general familiarity with the kind of portrait images used in the present study, participants were asked to respond to the question, ‘How familiar are you with the kind of images you just assessed? In other words, how often do you encounter such images in everyday life?’ using a 7-point rating scale from 1 = ‘not familiar at all / I almost never encounter such images’ not to 7 = ‘very familiar / I encounter similar images very often’.

*Criteria for aesthetic assessments.* A matrix question assessed the relative importance of 15 different aspects when judging image beauty. Participants judged how important they found each aspect on a scale from 1 = ‘not at all’ to 7 = ‘very much’. The items were adapted from earlier research on art perception [70]. Some of the items referred to potential bottom-up, or basic, visual-perceptual aspects of image evaluations, whereas others referred more to more top-down, cognitive-evaluative aspects. In addition to completing the matrix question, participants were given an open response field and could briefly describe in their own words the aspects that influenced their decision to ‘keep’ a picture or give it a high rating.

*Image beauty in relation to object beauty.* Using two sets of Likert scale questions, we asked participants how important they found the beauty of an image in comparison to the beauty of the depicted object when thinking about whether or not to keep an image in their ‘image collection’ depending on the image genre. Participants first rated, using a 7-point scale (1 = not at all to 7 = very much), how important they found image beauty for seven common image genres: 1. Faces/portraits of others, 2. Selfies, 3. Groups (friends/family), 4. Food, 5. Landscape/nature, 6. Urban or street scenes, and 7. Holiday pictures. Next, using the same format, they rated how important they found the beauty of the depicted object for their decision for the same seven image genres.

*Photography expertise.* To assess our participants' photography expertise which could also play a role in aesthetic evaluations [71], we first asked them to indicate how often they usually take photos of the seven different image genres in a matrix question. They rated the frequency for each genre on a 7-point scale with the options 1. Never, 2. Very rarely, 3. A few times a year, 4. About once a month, 5. About once a week, 6. Several times a week, and 7. Daily or almost daily. After that, they indicated if they would describe themselves as amateur photographers (yes/no) and whether they had anything to do with photography professionally (yes/no). Finally, we asked them to indicate which devices or cameras they used for photography, with the options smartphone, action camera, digital compact camera, DSLR/DSLM, analogue camera with film, and instant camera. If they did not take photographs, they could select the response option ‘I do not take photographs’.

*Questions following the attractiveness ratings.* After completing the attractiveness rating, participants could, on a voluntary basis, indicate their socio-sexual orientation by either providing their own label in an open response field or by selecting one of five predefined options. Next, they indicated how well they could separate the dimensions of image beauty and person attractiveness on a scale from 1 = ‘I could not separate this (image beauty was dependent on the attractiveness of the person)’ to 7 = ‘I was able to separate it very well (image beauty was independent of the attractiveness of the person)’. In an open field, we asked them to describe any aspects that played a stronger role in the assessment of attractiveness compared to the assessment of image beauty.

*Assumed image sources.* To assess whether participants thought that the pictures represented real persons or whether they were artificially generated, participants were also presented with the question ‘What sources do you think the images you judged came from?’, together with an open response field.

##### 2.4.2.5. Visual Aesthetics Sensitivity Test

To assess individual differences in aesthetic sensitivity, we used a short form of the Visual Aesthetics Sensitivity Test (VAST; [69]). The version used here included 25 monochromatic image pairs that were selected based on their psychometric properties from the original set [65]. Each image pair consisted of two similar images, one of which had a better design, for example, it was more harmonious, better balanced, or its elements were better arranged. The instructions were adapted from the original version and translated into German. Each trial started with the presentation of a fixation cross for 1s. After that, the image pair appeared, and participants chose, using the left and right arrow keys, the image that they considered better. Throughout the task block, the question ‘Which design is better?’ was also displayed at the bottom of the screen as a reminder of the task, along with an illustration of the response keys. The number of correct answers was used as a measure of individual aesthetic sensitivity.

#### 2.4.3. Data analysis

Rating and swiping task data were analysed using repeated measures analyses of variance (ANOVAs) with Type III sums of squares. Because not all participants use rating scales in the same way, and we also wanted to relate individual variability in the rating data to other variables that differed between participants, we standardized (z-transformed) all rating responses within participants. In general, we assumed *p* values below an *α* of 0.05 as statistically significant. All data analyses were performed using R 4.1.2 [72].

## 3. Results

### 3.1. Manipulation check

**Figure 3.**
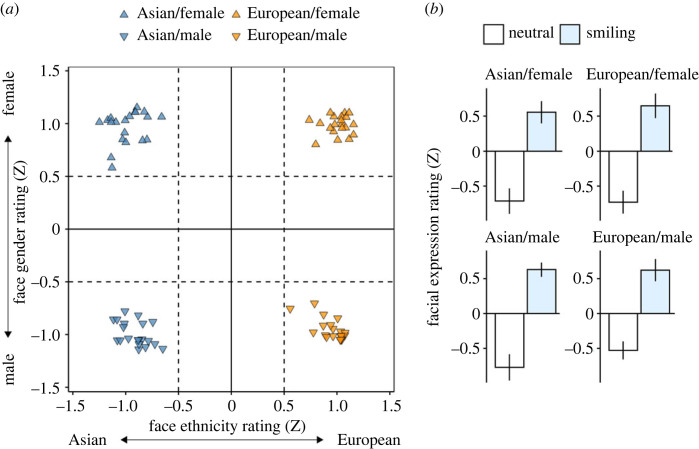
Manipulation checks based on data from the pilot experiment with *N* = 12 independent raters. (*a*) Data on the categorization of the stimuli into Asian/European or male/female faces. For each of the four face categories, 20 different faces were used in the final stimulus set for which the average standardized ratings on both dimensions exceeded ±0.5, suggesting that the categories were well separated. (*b*) Emotional expression ratings for the final stimulus set. For each of the included categories, the expected differences between smiling and neutral versions were clearly confirmed. We therefore assume that participants could reliably discriminate the subtle differences in facial expression, and that the stimulus set was therefore well suited for the main experiments.

Data from the pilot experiment confirmed that our manipulation of emotional expression and assignment of stimuli to the gender and ethnicity categories worked as intended. When smiling and neutral versions of the same face identities were presented side by side, the more positive version was correctly selected in, on average 91.4% (*s.e.* = 0.82) of the cases, which was, as expected, significantly better than chance, *t*_79_ = 50.56, *p* < 0.001. When the faces were presented individually and rated in terms of their emotional expression, the standardized ratings were significantly higher for the smiling (*M* = 0.61, *s.e.* = 0.04) than for the neutral version (*M* = −0.69, *s.e* = 0.04), *t*_79_ = 37.57, *p* < 0.001. Critically, the 80 faces used in the main experiments were unambiguously assigned to the categories of male/female and Asian/European, as none of the faces included in the final stimulus set had a standardized *Z*-score on either of the dimensions in the range of smaller than ±0.5, suggesting that the categories were well separated (figure 3).

### 3.2. Spontaneous aesthetic preferences as measured by the swiping task

**Figure 4.**
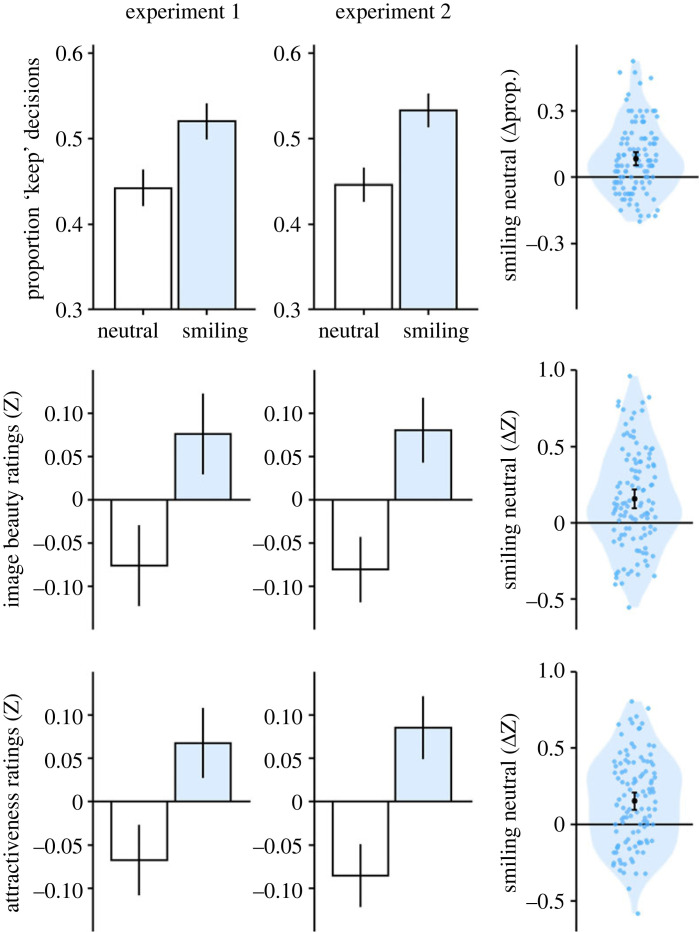
The positive effect of emotional expression in the three experimental tasks in Experiments 1 and 2 (first and second columns), and its variability across participants (i.e. the difference between conditions with smiling minus conditions with neutral faces). Bar graphs and error bars represent means ± 1 *SE*. Data points represent individual participants. (*a*) Portraits with a smiling expression were ‘kept’ significantly more often than faces with a neutral expression in both Experiments 1 and 2. (*b*) Portraits with a smiling expression were consistently rated as more beautiful than portraits with a neutral expression in both Experiments 1 and 2. (*c*) The depicted persons were also consistently rated as more attractive in both experiments when they had a smiling expression than when they had a neutral expression.

**Figure 5.**
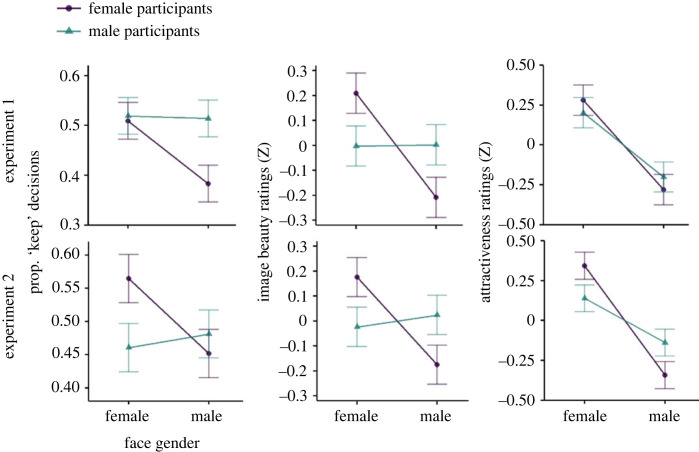
Interaction of Participant Gender × Face Gender in the three experimental tasks. First column: in spontaneous aesthetic decisions, female participants differentiated by face gender whereas male participants did not differentiate by female and male faces. Second column: for female participants, face gender also played a role in the assessment of image beauty, whereas male participants did not differentiate according to face gender when rating image beauty. Third column: when rating the attractiveness of the depicted person, both female and male participants rated female face images as more attractive than male faces. The interaction of Participant Gender × Face Gender was only significant in Experiment 2, as female participants in this sample still differentiated more strongly by face gender compared with male participants.

The results of the ANOVA of the swiping data are summarized in table 1. In both experiments, the analysis revealed a clear main effect of facial expression: for photos with a smiling facial expression, a ‘keep’ decision was made significantly more often compared with photos with a more neutral facial expression (figure 4, top row). The size of this smile effect did not differ between Experiment 1 and Experiment 2, *t*_110_ = −0.31, *p* = 0.756. Furthermore, in both experiments we observed an interaction of Participant Gender × Face Gender, with female participants showing a much stronger selectivity for male faces (figure 5). In addition, Experiment 1 also revealed a main effect of Face Gender, with pictures showing female faces being preferred over pictures showing male faces. Experiment 1 also yielded a three-way interaction of Participant Gender × Face Gender × Face Expression (see electronic supplementary material, S2). However, these last two results were not replicated in the separate sample of Experiment 2, so they may not be particularly robust (see also effect sizes, table 1).

**Table 1.** ANOVA of swiping decisions in Experiment 1 and Experiment 2.

effect	experiment 1			experiment 2		
	*F*	*p*	*η*^2^	*F*	*p*	*η*^2^
participant gender	3.97	0.051	0.07	0.77	0.383	0.01
face gender	6.35	0.015*	0.11	3.23	0.078	0.06
face ethnicity	0.38	0.540	0.01	3.15	0.081	0.06
face expression	12.92	<0.001***	0.19	18.36	<0.001***	0.25
participant gender × face gender	5.44	0.023*	0.09	6.77	0.012*	0.11
participant gender × face ethnicity	0.04	0.837	0.00	0.02	0.889	0.00
participant gender × face expression	0.56	0.456	0.01	0.01	0.913	0.00
face gender × face ethnicity	0.37	0.545	0.01	0.09	0.762	0.00
face gender × face expression	1.06	0.308	0.02	0.87	0.356	0.02
face ethnicity × face expression	2.86	0.097	0.05	0.03	0.854	0.00
participant gender × face gender × face ethnicity	2.32	0.134	0.04	2.15	0.149	0.04
participant gender × face gender × face expression	5.67	0.021*	0.09	0.17	0.678	0.00
participant gender × face ethnicity × face expression	1.33	0.254	0.02	0.73	0.398	0.01
face gender × face ethnicity × face expression	0.42	0.519	0.01	3.77	0.057	0.07
participant gender × face gender × face ethnicity × face expression	3.22	0.078	0.06	0.34	0.561	0.01

*Note*. * *p* < 0.05, ** *p* < 0.01, *** *p* < .001. *df* = (1,54).

### 3.3. Ratings of image beauty after longer viewing (6 s)

The ANOVA of image beauty ratings (table 2) also revealed a clear main effect of facial expression, with pictures showing a smiling expression being rated, on average, more beautiful compared with pictures showing a more neutral expression (figure 4, middle row). The size of the effect did not differ between Experiments 1 and 2, *t*_110_ = **−**0.15, *p* = 0.885. The analysis further yielded an interaction of Participant Gender × Face Gender, with a pattern similar to the swiping task (figure 5): female participants assigned, on average, lower ratings to pictures showing male faces compared with pictures showing female faces. By contrast, male participants did not differentiate according to face gender. The analysis also confirmed a main effect of face gender in both experiments, with on average higher ratings of female versus male faces. Lastly, only Experiment 1 yielded an interaction of Participant Gender × Facial Expression, with female participants showing a stronger effect of facial expression compared with male participants. Note however, that because in both experiments the between-participant variability in the smile effect was relatively high, this finding might be attributed to a sampling effect (in a control analysis across data from both experiments, the interaction of Participant Gender × Facial Expression was not significant, see electronic supplementary material, S3).

**Table 2.** ANOVA of image beauty ratings in Experiment 1 and Experiment 2.

effect	experiment 1			experiment 2		
	*F*	*P*	*η*^2^	*F*	*p*	*η*^2^
participant gender	0.00	>0.999	0.00	0.00	>0.999	0.00
face gender	13.12	<0.001***	0.20	7.48	0.008**	0.12
face ethnicity	0.05	0.827	0.00	1.16	0.286	0.02
face expression	11.51	0.001**	0.18	17.36	<0.001***	0.24
participant gender × face gender	13.78	<0.001***	0.20	12.96	<0.001***	0.19
participant gender × face ethnicity	0.85	0.361	0.02	0.10	0.759	0.00
participant gender × face expression	8.27	0.006*	0.13	0.51	0.476	0.01
face gender × face ethnicity	1.94	0.169	0.03	0.36	0.550	0.01
face gender × face expression	1.62	0.209	0.03	1.30	0.259	0.02
face ethnicity × face expression	1.70	0.197	0.03	0.98	0.327	0.02
participant gender × face gender × face ethnicity	0.02	0.879	0.00	0.01	0.938	0.00
participant gender × face gender × face expression	0.00	0.971	0.00	0.42	0.521	0.01
participant gender × face ethnicity × face expression	2.61	0.112	0.05	0.01	0.927	0.00
face gender × face ethnicity × face expression	1.45	0.233	0.03	3.08	0.085	0.05
participant gender × face gender × face ethnicity × face expression	0.39	0.537	0.01	0.00	0.946	0.00

N*o*te. * *p* < 0.05, ** *p* < 0.01, *** *p* < 0.001. *df* = (1,54).

### 3.4. Ratings of person attractiveness

**Figure 6.**
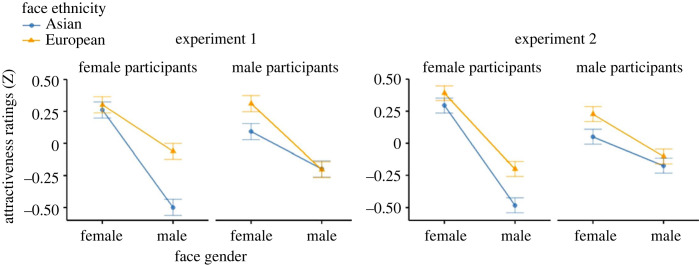
The three-way interaction of Participant Gender × Face Gender × Face Ethnicity which was replicated across both experiments and showed a qualitatively consistent pattern: female participants differentiated for male but not for female faces according to ethnicity of the face and rated European-looking faces as more attractive. This pattern was reversed for male participants, who differentiated by face ethnicity only for female but not for male faces and rated European-looking female faces as more attractive compared with Asian-looking female faces.

The ANOVA results of the attractiveness ratings (table 3) revealed patterns that were partly distinct from the swiping and image beauty tasks. Once again, the analysis confirmed a strong main effect of facial expression: on average, persons who smiled on the picture were rated as more attractive compared with persons with a more neutral expression. The size of the smile effect did not differ between Experiments 1 and 2, *t*_110_ = **−**0.64, *p* = 0.521. Crucially, face gender and face ethnicity played a stronger role in the attractiveness task, culminating in a significant three-way interaction of Participant Gender × Face Gender × Face Ethnicity in both experiments (figure 6). Female participants differentiated by ethnicity for male faces, and rated European male faces as more attractive than Asian male faces, whereas they did not differentiate by ethnicity for female faces. The male participants showed the exact opposite pattern, and preferred European ethnicity only for female faces but not for male faces. These results were consistent across both experiments, suggesting that participants indeed differentiate between aspects of the portrait pictures depending on whether they evaluate image aesthetics or the attractiveness of the person, and gender and ethnicity appear to be specifically taken into account when evaluating attractiveness.

**Table 3.** ANOVA of attractiveness ratings in Experiment 1 and Experiment 2.

effect	experiment 1			experiment 2		
	*F*	*p*	*η*^2^	*F*	*p*	*η*^2^
participant gender	0.00	>0.999	0.00	0.00	>0.999	0.00
face gender	51.66	<0.001***	0.49	65.07	<0.001***	0.55
face ethnicity	8.33	0.006**	0.13	6.83	0.012*	0.11
face expression	10.40	0.002**	0.16	20.87	<0.001***	0.28
participant gender × face gender	1.40	0.242	0.03	11.68	0.001**	0.18
participant gender × face ethnicity	1.24	0.271	0.02	0.30	0.586	0.01
participant gender × face expression	0.22	0.637	0.00	0.29	0.589	0.01
face gender × face ethnicity	1.88	0.176	0.03	0.52	0.473	0.01
face gender × face expression	2.00	0.163	0.04	2.23	0.141	0.04
face ethnicity × face expression	1.55	0.218	0.03	0.80	0.376	0.01
participant gender × face gender × face ethnicity	24.53	<0.001***	0.31	6.35	0.015*	0.11
participant gender × face gender × face expression	0.24	0.628	0.00	0.15	0.703	0.00
participant gender × face ethnicity × face expression	1.87	0.177	0.03	0.24	0.629	0.00
face gender × face ethnicity × face expression	0.29	0.593	0.01	0.97	0.330	0.02
participant gender × face gender × face ethnicity × face expression	0.16	0.694	0.00	0.01	0.929	0.00

*Note*. * *p* < 0.05, ** *p* < 0.01, *** *p* < 0.001. *df* = (1,54).

### 3.5. Correlation of image beauty and attractiveness ratings

We determined the correlations of image beauty and attractiveness ratings obtained for the same images within each participant and applied a Fisher z-transformation to calculate the average correlations across participants. On average, these correlations amounted to *z* = 0.39 (*s.e.* = 0.02) in Experiment 1, which corresponds to *r*_54_ = 0.37. In Experiment 2, the average correlation was *z* = 0.43 (*s.e.* = 0.07) or *r*_54_ = 0.41. The size of the correlations did not differ between experiments, *t*_110_ = **−**0. 53, *p* = 0.597. It is noteworthy that participants' subjective ratings of how well they could separate between the two aspects (on a scale from 1 = not well to 7 = very well), were negatively related to the actual correlation between their image beauty and attractiveness ratings, with *r*_54_ = **−**0.29, *p* = 0.032 in Experiment 1 and *r*_54_ = **−**0.37, *p* = 0.004 in Experiment 2, respectively. To summarize, although we observed positive correlations of image beauty and attractiveness ratings, they were far from perfect, suggesting that participants differentiated between these aspects. The participants were also fairly accurate in assessing how well they could personally separate between the two aspects.

### 3.6. Criteria used in aesthetic evaluations

**Figure 7.**
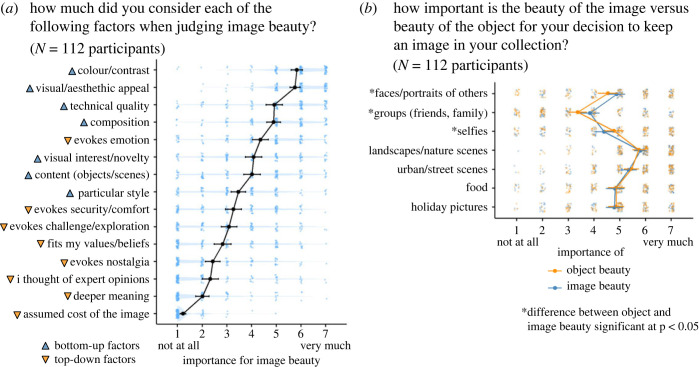
Results of standardized questionnaire items. (*a*) Subjective importance of different bottom-up or top-down factors for image beauty judgements. Items were adapted from Pelowski *et al*. [70]. (*b*) Assessments of the relative importance of beauty of the image versus beauty of the object for seven common image genres. Significant differences (from two-tailed *t*-tests) were obtained for the three categories containing faces. Background data points represent responses from individual participants. Lines represent means and standard errors across participants.

To understand the participants' aesthetic experience more comprehensively, we asked them which dimensions they considered particularly important for their evaluations and decisions. Figure 7*a* shows that especially image-related aspects such as colour/contrast, visual appeal, composition, were rated as particularly important by most participants. But interestingly, emotion, as a factor that could be considered more top-down or viewer-dependent (cf. [70]), was, on average, also ranked relatively high, whereas other top-down factors were considered less important. Also interesting, regarding the relative importance of image beauty in comparison to object beauty, participants mostly differentiated between these aspects in image categories that contain faces or persons (figure 7*b*). This suggests that in the genre of portraits, image beauty might often be considered as a separate aspect, in addition to the attractiveness of the depicted persons.

**Figure 8.**
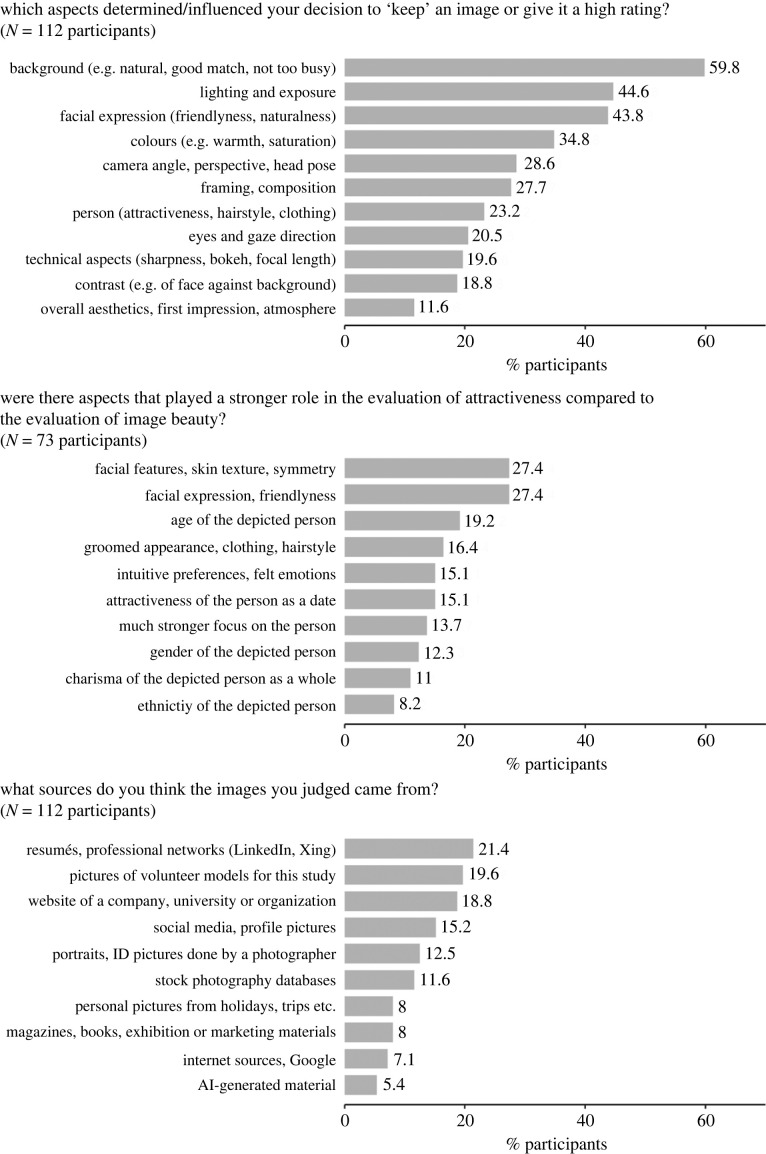
Analysis of open responses provided by the participants. Data from Experiments 1 and 2 were combined for this analysis. Similar responses or mentions were grouped into categories. Depending on the content, individual participants' responses could be assigned to more than one category. Values indicate the percentage of participants whose responses fell in the respective category. Categories are sorted by frequency of mentions. (*a*) Responses to the question ‘Which aspects determined/influenced your decision to ‘keep’ an image or give it a high rating?’. This was a required response field, with percentages based on *N* = 112 participants. (*b*) Responses to the question, ‘Were there aspects that played a stronger role in the evaluation of attractiveness compared to the evaluation of image beauty?’. This was an optional response, with percentages based on *N* = 73 participants. (*c*) Responses to the question, ‘What sources do you think the images you judged came from?’. This was a required response field, with percentages based on *N* = 112 participants.

We also asked the participants to describe, in their own words, the aspects that were of particular importance for their aesthetic evaluations and attractiveness judgements. For the decision to keep an image or give it a high image beauty rating, aspects such as background, exposure, colours, camera angle and framing were frequently mentioned. Interestingly, in terms of image content, facial expression was also among the frequent mentions. In comparison, for the evaluation of the attractiveness of the person, participants mentioned a range of additional person-related aspects which were not described as important for the evaluation of image beauty (figure 8, first and second plot). Regarding the presumed origin of the images (figure 8, third plot), participants mentioned diverse possible sources, but only six out of 112 participants suspected the pictures to be possibly AI-generated, indicating that the material appeared rather convincing and natural to the participants.

## 4. Discussion

In the present study, we employed image-, viewer- and context-related variables and tested if a subtle change in facial expression reliably increases the aesthetic value of portraits.

### 4.1. Emotional expression is a decisive feature for aesthetic evaluations of portraits

We found that portraits with a subtle smile were generally considered more beautiful than portraits with a more neutral expression. This suggests that participants based their aesthetic judgements on the psychologically relevant feature of emotional expression, even during spontaneous and intuitive aesthetic judgements which we investigated here, for the first time, using a novel type of ‘swiping’ task that mimics the type of rapid and intuitive decisions that many people often make when browsing through a stack of pictures on their mobile phones [1]. As expected, the emotional expression also influenced how attractive participants found the depicted person, replicating earlier findings that argued for a link between positive emotional expression and attractiveness (e.g. [25–27]).

Additional analyses of individual differences (electronic supplementary material S4) revealed that not all participants were equally sensitive to this feature. Specifically, we found that stronger expertise in photography was negatively associated with the effectiveness of emotional expression manipulation. Interestingly, in a previous study, Mulas *et al*. [71] compared photography experts with non-experts and found, similarly to the present study, that a positive emotional expression increased picture evaluations in non-experts only. These consistent results suggest that experts probably focus on a broader set of image aspects and compare them to their own, more elaborated, image beauty standards which they acquired over time. Conversely, the emotional expression of the depicted person seems particularly important for raters who are less familiar with (portrait) photography. As a psychologically and socially highly relevant feature, the facial expression may be processed and perceived effortlessly (cf. [73]), which could explain why people who have less expertise in photography rely more strongly on this easily accessible feature because it also plays an important role in their everyday life.

### 4.2. Manipulating emotional expression to enhance the aesthetic value of a portrait

In our study, we applied a rather subtle manipulation of emotional expression to change the appearance of the original images. Nevertheless, the manipulation had a robust effect on the participants' evaluations that could be consistently replicated across two experiments and different tasks. It is worth mentioning that the portraits used in the present study were generated and manipulated using modern image-processing tools [68]. However, the responses of our participants suggested that they perceived them as actual photographs of real people, and only 6 out of 112 participants expressed the suspicion that the material could be artificially generated. This illustrates how advanced the currently available image processing tools are and how convincingly they can alter the emotional expression in a portrait.

Notably, we changed the emotional expression by changing only the mouth region of the faces, and the participants were sensitive to this very subtle and local manipulation of facial expression. Therefore, this kind of subtle post-processing might also be useful for improving the perceived aesthetic quality of portrait images in practice. Current mobile phones already allow for certain aspects of images to be post-processed on the fly without requiring any special expertise or effort on the part of the user. For example, image beautification filters can smoothen the skin texture, and thus enhance the perceived beauty, making ‘digital make-up’ part of the image processing [74]. Similarly, bokeh filters selectively blur the background to simulate the effect of shallow depth of field that is usually achieved with professional camera equipment and large aperture portrait lenses (e.g. [75]). Our data suggest that adjusting the emotional expression of the portrayed person could similarly improve the perceived aesthetic quality of the image. Because even a slight adjustment of the expression had replicable benefits on aesthetic evaluations, a practical implementation of such a filter could also embrace the principle of ‘less is more’, and avoid overprocessing the images as this could result in an ‘uncanny valley’ effect where the faces could appear unnatural if the manipulation is too strong (cf. [76]).

### 4.3. Independence of image beauty and attractiveness assessments

An important question we addressed in the current study was whether participants are able to judge the beauty of an image and the attractiveness of the depicted person as independent aspects. This is relevant because an artistic portrait might be perceived as beautiful even if the depicted person is not perceived as attractive [20]. In everyday life, the distinction between image beauty and person attractiveness probably plays a large role. Especially in the case of persons with whom one is more familiar, the experience and acquaintance with this person could influence attractiveness judgements (cf. [77]), whereas different pictures of the same person could be perceived as more or less beautiful.

Even though emotional expression influenced the judgements in all three tasks in the same direction, our analyses showed that the correlations between the tasks were not perfect and participants seemed to differentiate between the dimensions of image beauty and person attractiveness. Whether the assessments of the two dimensions were assessed in the same (Experiment 1) or different (Experiment 2) task blocks did not significantly change the extent to which participants differentiated between them. The mentions of the aspects that participants found relevant for the different tasks suggested that participants focused their attention on different features, depending on the task: whereas topics such as image background, exposure, colours, camera angle or composition were frequently mentioned as critical when evaluating image beauty, most participants mentioned features directly related to the depicted persons and their characteristics when evaluating attractiveness. Noteworthily, however, in both tasks, the person's facial expression was very frequently mentioned as a decisive feature, which further underscores its importance.

The remaining positive correlation between the ratings of image beauty and attractiveness suggests that the two dimensions are not completely independent. However, a certain positive correlation is to be expected due to the high standardization of the stimulus material. For independent dimensions, one would need an image set in which attractive individuals are poorly photographed and unattractive individuals are well photographed. Such a situation is quite conceivable, and we would suspect that in this case the correlation found would be much lower. Also due to the high standardization of the image material, we suspect that the current study provides a rather conservative estimate of the independence of the two dimensions. The present portraits were similar in terms of framing, apparent distance from the person depicted. This high degree of standardization, of course, also meant that the set of image features that typically differed between portraits was limited to some extent, and we suspect that the correlation between evaluations of image beauty and the attractiveness of the person depicted would further reduce with more a variable image set.

### 4.4. The role of gender in the assessment of portraits

Consistent with previous research on facial attractiveness, our analyses revealed interactions between the gender of the participant, and the gender of the depicted person [16,17,46]. However, in the present study, the nature of these interactions depended on the particular task context. First, female and male participants showed a different pattern in spontaneous and explicit judgements of image beauty: men did not differentiate between portraits of female and male persons, whereas women showed a much stronger selectivity for images showing male persons, which were much more often ‘deleted’ and received on average lower ratings than images showing female persons. By contrast, when rating attractiveness, the results pattern of male and female participants was much more consistent. The general preference for female faces shown in both studies is perhaps not surprising, as previous research also found that female morphological features are rated as more attractive (cf. [50]).

### 4.5. Face ethnicity effects in attractiveness ratings

An interesting yet originally unpredicted result in the present study was participants' consideration of face ethnicity when assessing attractiveness of opposite-gender faces. Female participants rated male European faces as more attractive compared to their male Asian counterparts but did not differentiate female faces based on their ethnicity. Conversely, male participants exhibited a reversed effect: only for female faces, they rated the European exemplars as more attractive than the Asian ones. Notably, this interaction effect of Participant Gender × Face Gender × Face Ethnicity was replicated with an independent participant sample in Experiment 2. For one, this robust result provides further evidence that participants differentiate between the dimensions of image beauty and person attractiveness, because this interaction did not occur in either of the other two tasks. However, due to its potential ethical implications, we believe it is important to reflect on this observation in more detail.

To start with, because we conducted our study in Europe, our participants might have exhibited an own-race bias in their attractiveness evaluations. The influence of unconscious racial bias on perception, cognition, and behaviour is well-documented across various contexts [78]. Importantly, such biases can manifest without necessarily indicating underlying racist attitudes. Individuals genuinely committed to treating all people equally could still harbour unconscious biases that inadvertently shape their judgements, memories and perceptual processes (e.g. [79]). Crucially, the generated portraits used in our study did not depict real people, and participants lacked any contextual knowledge about them. Thus, the judgements were rooted solely in visual features, and our participants may have primarily based their evaluations on visual features that allowed them to categorize the faces in the absence of further contextual information. Thus, by making the face stimuli easily discriminable as Asian versus European, we may have unintentionally fostered an own-race bias in the attractiveness task. Yet, other aspects of the results speak against the idea of a general own-race bias in the present participant samples. On the one hand, the attractiveness data show that Asian female faces were always rated higher in attractiveness than European male faces. However, we did not observe any effects of face ethnicity on explicit aesthetic evaluations of image beauty or on the intuitive swiping decisions, suggesting that the preference for European opposite-gender faces was strictly confined to attractiveness evaluations. Thus, participants apparently did not consider face ethnicity as an important variable in all their evaluations.

When assessing attractiveness, participants might generally understand it as attractiveness in the context of potential mate choice. Prior research in this field offers various clues that could explain the current attractiveness preference for opposite-gender European faces. For example, familiarity often seems to play a role in judgements of attractiveness [80]. Because our experiments were conducted in Europe, the phenotypic features of these faces are closer to the majority of persons with whom our participants usually interact [51,54,55]. Although classic mere exposure effects [81] could play a certain role, the rather specific pattern of results suggests that more complex processes are at work which could include, for example, ‘imprinting’ to opposite-gender parental traits [82], or prior positive experiences with similar looking individuals [83,84]. Based on the latter, we could speculate that participants may draw comparisons between the assessed faces and their previous partners or individuals they have found attractive or trustworthy. Within a European sample, the European-looking opposite-gender faces might simply resemble their own partners, people that they found attractive, or were romantically involved with, to a stronger degree. To follow up on this open question, future research could use the present set of stimuli and collect evaluations in a comparable Asian sample. If the underlying mechanisms generalize across cultural contexts, we should anticipate a mirrored pattern of results, with attractiveness ratings for Asian-looking opposite-gender faces surpassing those for European-looking ones.

### 4.6. Understanding the aesthetics of mobile phone photography

The current study is the first to use the theoretical taxonomy of Leder *et al*. [1] to investigate the psychological and contextual factors that make up a good photograph. We tested central predictions of the model and collected additional measures to better understand how viewers make decisions about the aesthetics of portrait photographs. Compared to other image categories, portraits or images of faces occupy a special position in today's media [74]. Not only are they among the most widely circulated images, but research also suggests that images of faces generate a particularly high level of interest and, for example, generate more comments and likes on social media than images of other categories [3]. Our current results suggest that emotional expression is a central and psychologically relevant feature of portrait images, and even a subtle change in emotional expression changes which pictures are spontaneously and intuitively perceived as more beautiful. Our results also revealed that different aspects of the picture are taken into account depending on the current task context. Whereas our current study focused on portrait photography, as one of the most widespread image genres today, future research should extend this promising approach, especially the comparison of aesthetic preferences in spontaneous versus more explicit evaluation tasks, to better understand the aesthetic experience of other photo genres.

## 5. Conclusion

A subtle manipulation of the facial expression in a portrait increased the perceived aesthetic value of the image. The benefits of ‘putting a smile on a face’ are robust and already affect rapid and spontaneous decisions, when images are only briefly viewed. Interestingly, this positive ‘smile effect’ was independent of contextual variables such as the gender of the participant, the viewing task, or the gender or ethnicity of the face and therefore seems to reflect a rather universal preference, although its strength weakened with the viewer's photographic expertise. Our results illustrate that current image processing tools can reliably manipulate psychologically important variables such as the emotional expression to improve the perceived beauty of the resulting image. Post-processing a person's emotional expression might seem like a radical break from the idea of photography as a snapshot of reality. However, facial expression is ultimately just another, albeit psychologically particularly important image feature which, similar to exposure or skin texture, could be adjusted by a modern digital camera or mobile phone to maximize the impact of the resulting image. What probably matters for most users of (portrait) photography is not whether the resulting image is a realistic depiction of the moment in which it was taken, but whether it matches the way they would like to remember the moment and share it with others.

## Data Availability

Data and R-code to reproduce the analyses are available through the Open Science Framework at https://osf.io/9f74y/. Additional analyses and data are provided in electronic supplementary material [85].
